# Identification of the pivotal role of SPP1 in kidney stone disease based on multiple bioinformatics analysis

**DOI:** 10.1186/s12920-022-01157-4

**Published:** 2022-01-11

**Authors:** Sen-Yuan Hong, Qi-Dong Xia, Jin-Zhou Xu, Chen-Qian Liu, Jian-Xuan Sun, Yang Xun, Shao-Gang Wang

**Affiliations:** Department of Urology, Tongji Hospital, Tongji Medical College, Huazhong University of Science and Technology, Wuhan, China

**Keywords:** Kidney stone disease, Weighted gene co-expression network analysis, Hub genes, SPP1, Macrophages

## Abstract

**Background:**

Kidney stone disease (KSD) is a multifactorial disease involving both environmental and genetic factors, whose pathogenesis remains unclear. This study aims to explore the hub genes related to stone formation that could serve as potential therapeutic targets.

**Methods:**

Based on the GSE73680 dataset with 62 samples, differentially expressed genes (DEGs) between Randall’s plaque (RP) tissues and normal tissues were screened and weighted gene co-expression network analysis (WGCNA) was applied to identify key modules associated with KSD. Gene Ontology (GO) and Kyoto Encyclopedia of Genes and Genomes (KEGG) enrichment analysis were performed to explore the biological functions. The protein–protein interaction (PPI) network was constructed to identify hub genes. Meanwhile, CIBERSORT and ssGSEA analysis were used to estimate the infiltration level of the immune cells. The correlations between hub genes and immune infiltration levels were also investigated. Finally, the top hub gene was selected for further GSEA analysis.

**Results:**

A total of 116 DEGs, including 73 up-regulated and 43 down-regulated genes, were screened in the dataset. The red module was identified as the key module correlated with KSD. 53 genes were obtained for functional enrichment analysis by taking the intersection of DEGs and genes in the red module. GO analysis showed that these genes were mainly involved in extracellular matrix organization (ECM) and extracellular structure organization, and others. KEGG analysis revealed that the pathways of aldosterone-regulated sodium reabsorption, cell adhesion molecules, arachidonic acid (AA) metabolism, and ECM-receptor interaction were enriched. Through PPI network construction, 30 hub genes were identified. CIBERSORT analysis revealed a significantly increased proportion of M0 macrophages, while ssGSEA revealed no significant differences. Among these hub genes, SPP1, LCN2, MMP7, MUC1, SCNN1A, CLU, SLP1, LAMC2, and CYSLTR2 were positively correlated with macrophages infiltration. GSEA analysis found that positive regulation of JNK activity was enriched in RP tissues with high SPP1 expression, while negative regulation of IL-1β production was enriched in the low-SPP1 subgroup.

**Conclusions:**

There are 30 hub genes associated with KSD, among which SPP1 is the top hub gene with the most extensive links with other hub genes. SPP1 might play a pivotal role in the pathogenesis of KSD, which is expected to become a potential therapeutic target, while its interaction with macrophages in KSD needs further investigation.

**Supplementary Information:**

The online version contains supplementary material available at 10.1186/s12920-022-01157-4.

## Introduction

Kidney stone disease (KSD) is one of the most common urological diseases worldwide with high incidence and recurrence rates, which contributes to a huge burden on the medical and health care system [1–3]. The most common type of stones is calcium oxalate (CaOx) mixed with calcium phosphate (CaP), accounting for approximately 80%, followed by struvite, uric acid, and cysteine [1, 4]. The most widely accepted theory of CaOx stone formation is Randall’s plaque (RP) theory, in which stone can grow on the renal papillary surface attached to interstitial apatite deposits called RPs [5, 6]. Although major advances in surgical techniques have greatly improved the effectiveness of stone removal, the exact mechanisms of stone formation and recurrence after surgery remain unclear, making the development of effective medical drugs stagnant.

Previous studies have discovered that several genes and their coded proteins were strongly linked with KSD, such as UMOD, MGP, SPP1, and so on [7]. SPP1, also known as osteopontin (OPN), is a secreted pleiotropic glycoprotein with diverse physiological and pathological functions [8]. As an important modulator of biomineralization, SPP1 is thought to be involved in stone formation, since RP is considered as a form of pathological biomineralization [9]. But previous studies showed that SPP1 played a dual role in promoting or inhibiting crystallization [10]. Therefore, there has been little agreement on the role of SPP1 in the pathogenesis of KSD. In addition, SPP1 is a chemical attractant for macrophages, which could regulate immune response through macrophages infiltration [8]. Recently, macrophage polarization is confirmed to play an important role in the development of KSD [11]. However, no study has investigated the relationship between SPP1 and macrophages in KSD, which needs to be fully explored.

For researchers, renal RP tissue is the key to exploring the underlying molecular mechanisms of KSD. The microarray study of the available gene expression profile of RP tissue (GSE73680) has linked many genes and pathways to KSD, but the analysis is relatively simple and a great deal of information has not been extensively mined [12]. This study was designed to investigate the GSE73680 dataset in greater depth through multiple bioinformatic methods to explore potential pathogenic genes and therapeutic targets. This study will use WGCNA for the first time to explore key modules significantly correlated with KSD, screen hub genes in the protein–protein interaction (PPI) network, and evaluate immune infiltration levels in RP tissues by CIBERSORT and single-sample gene set enrichment analysis (ssGSEA). The correlations between hub genes and macrophages were also investigated. Finally, the top hub gene, SPP1, was selected for subsequent gene set enrichment analysis (GSEA).

## Methods

### Data collection and preprocessing

**Fig. 1:**
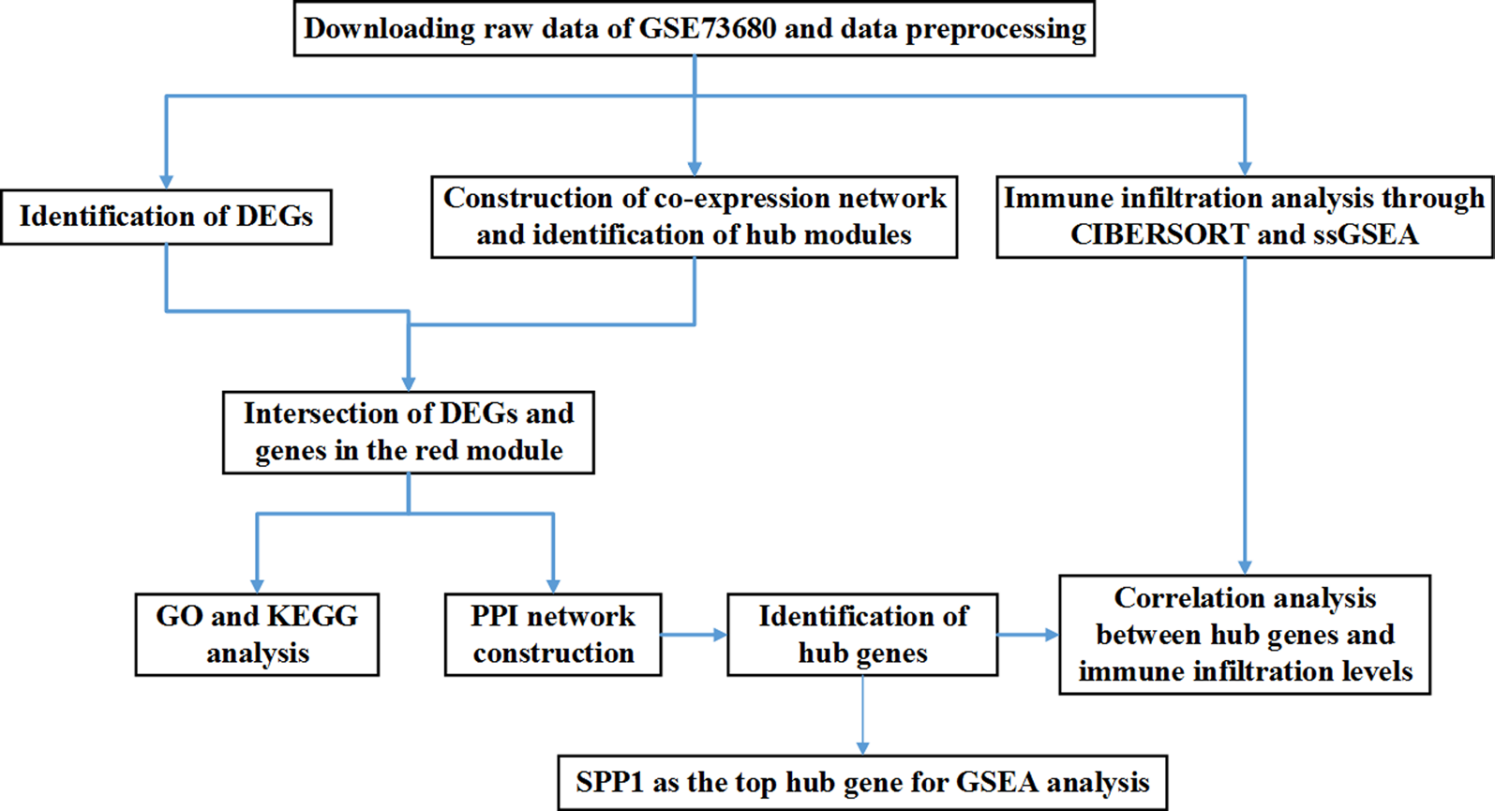
Flow chart of the whole procedures

The flowchart of the study is shown in Fig. 1. The code used to perform the whole process was presented in Additional file 1. KSD related datasets were retrieved from the Gene Expression Omnibus (GEO) database (http://www.ncbi.nlm.nih.gov/geo/) using “kidney stone” as a search keyword. Two datasets (GSE73680 and GSE117518) which collected transcriptome data from RP papillary tissues and normal renal papillary tissues were obtained. However, GSE117518 was excluded because of the small sample size (3 RP papillary tissues, 3 normal papillary tissues). Hence, GSE73680 was screened as an appropriate dataset for further analysis due to the relatively large sample size (29 RP papillary tissues and 33 normal papillary tissues).

The original raw data of each sample in GSE73680 was downloaded. As GSE73680 dataset was sequenced by Aglient microarray probe, the raw data was read by the “limma” package (version 3.48.3), the background of each sample was corrected by the “backgroundCorrect” function in “limma” package, and the data was normalized between different arrays by the “normalizeBetweenArrays” function in “limma” package [13]. The following steps were sequentially applied: removing the control probe, removing the probes that match no gene symbol, and removing the duplicated gene probes. Finally, the gene matrix was annotated according to the annotation files of the Agilent-039494 SurePrint G3 Human GE v2 8x60K Microarray 039381 (Probe Name version) for further analysis.

### Identification of differentially expressed genes (DEGs)

GSE73680 dataset enrolled 62 renal papillary tissue samples, including 29 RP papillary tissues from stone formers, 27 normal papillary tissues from stone formers, and 6 normal papillary tissues from control patients. The “limma” package was used to identify DEGs between 33 normal papillary tissues and 29 RP papillary tissues with the threshold of false discovery rate (FDR) < 0.05 and |log_2_ fold change (FC)| > 1 [13]. The volcano plot of DEGs was drawn by the “ggplot2” package (version 3.3.5), and the heatmap of DEGs was generated using the “pheatmap” package (version 1.0.12) in the R software (version 4.1.1).

### Construction of a weighted co-expression network and identification of hub modules

The “WGCNA” package (version 1.70.3) was applied to construct the co-expression networks and identify the disease-related hub modules [14]. First, the Pearson's correlation matrices were performed for all paired genes and a weighted adjacency matrix was constructed with the formula a_mn_ = |c_mn_|^β^ (c_mn_ = Pearson's correlation between gene m and gene n; a_mn_ = adjacency between gene m and gene n). Next, the soft-threshold power value that emphasizes strong correlations between genes and penalizes weak correlations was calculated and a suitable parameter β was screened to build a scale-free network. Then, the weighted adjacency matrix was transformed into a topological overlap measure (TOM) matrix, which could measure the network connectivity of a gene defined as the sum of its adjacency with all other genes for the network generation [15]. After that, average linkage hierarchical clustering was conducted to classify genes with similar expression profiles into the same gene modules according to the TOM-based dissimilarity measure with a minimum size of 50 for the gene dendrogram [16]. The correlation between module eigengenes and clinical traits was assessed by Pearson correlation test to identify the significant modules. The KSD-related module was selected with the highest coefficient square (R^2^) and the *P* value < 0.05.

### Functional and pathway enrichment analysis

Following the steps above, two gene lists were obtained, one for the DEGs, the other one for the most KSD-related gene modules. Subsequently, the intersection of these two gene lists was taken to identify the KSD-related genes and a Venn diagram was constructed via the “VennDiagram” package (version 1.7.1). The Gene Ontology (GO) and the Kyoto Encyclopedia of Genes and Genomes (KEGG) pathways enrichment analysis were conducted to define the potential functions and pathways between these genes using the “org.Hs.eg.db” (version 3.10.0), “clusterProfiler” (version 3.14.3), “enrichplot” (version 1.6.1), and the “ggplot2” (version 3.3.5) packages in the R software [17, 18]. The GO terms of biological processes (BP), molecular functions (MF), and cellular components (CC) were respectively evaluated. Significant results were determined under the condition of adjusted *P* value < 0.05. The top ten terms were visualized if there were more than ten terms.

### Construction of protein–protein interaction (PPI) network and identification of hub genes

The PPI network of the KSD-related genes was analyzed with the STRING database (http://string-db.org/) and visualized using Cytoscape software (version 3.8.2). To identify hub genes, the sides of each node were estimated and the genes were sorted based on the rank of the connection number of each gene. The top 30 genes with the largest edges connected to them were selected as hub genes and the gene rank 1st was identified as the top hub gene for further GSEA analysis.

### Immune infiltration analysis through CIBERSORT and ssGSEA

For exploring the different infiltration degrees of immune cell types between RP tissues and normal tissues, the CIBERSORT algorithm was conducted to classify and quantify the abundance of 22 types of immune cells by R program [19]. The violin plot was generated using the “vioplot” package (version 0.3.7). Spearman correlation analysis was performed to examine the correlations between the hub genes and immune infiltrations. Meanwhile, the ssGSEA was used to estimate the infiltration levels of immune cells and immune-related functions in renal papillary tissues using the “GSVA” package (version 1.40.1) [20].

### Gene set enrichment analysis (GSEA)

SPP1, the most connected node, was identified as the top hub gene for GSEA analysis. To further explore the role of SPP1 in KSD, 29 RP papillary tissues were divided into low- and high-expression groups by the median expression value of SPP1. Then GSEA analysis was applied to compare the differential enhanced functions or pathways between low- and high-SPP1 expression groups by GSEA software (version 4.1.0) [21]. The c5.go.v7.4.symbols.gmt datasets in MsigDB were used as reference gene sets and GSEA analysis was performed according to default parameters [22]. The NOM *P* value < 0.05 was considered significant.

## Results

### Identification of DEGs

**Fig. 2:**
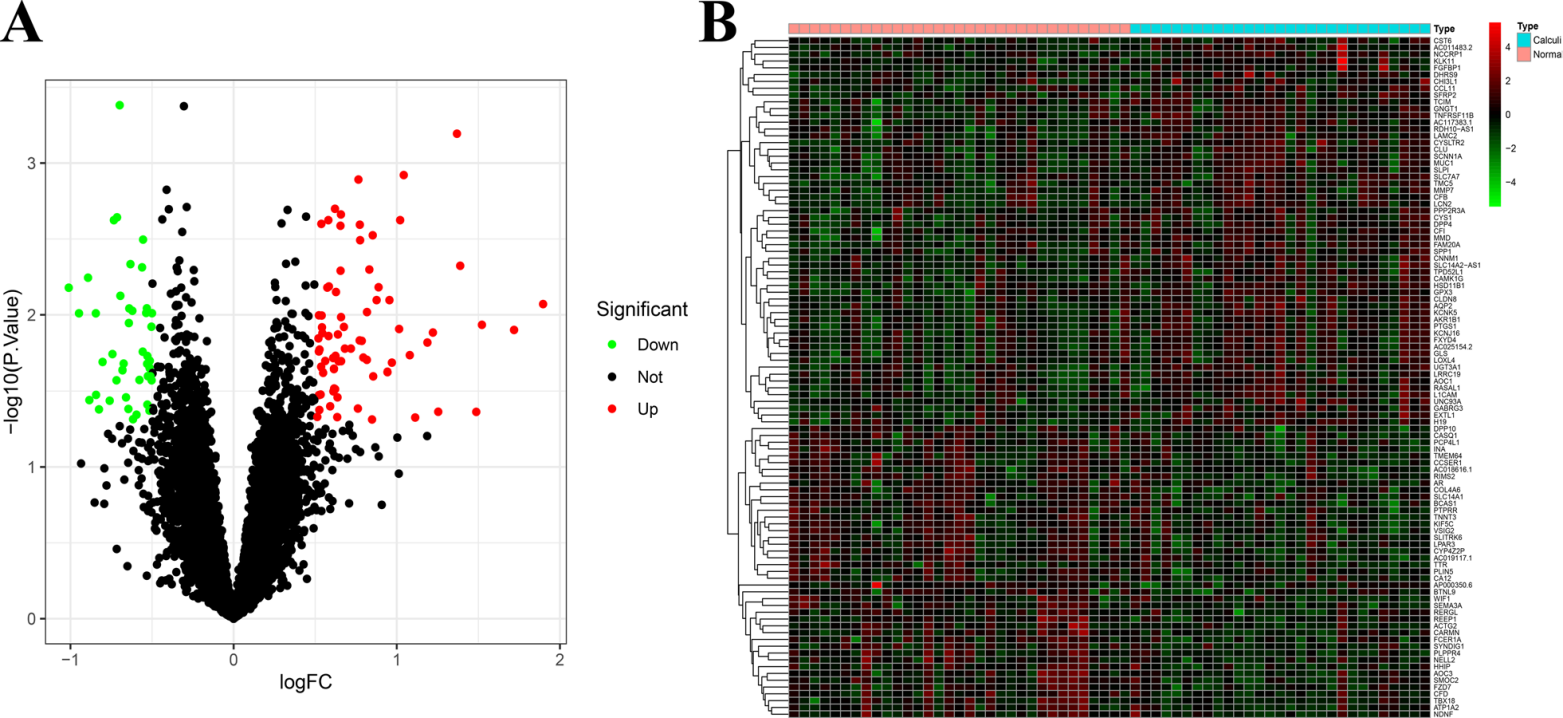
Identification of DEGs in GSE73680. **A** Volcano plot of all DEGs. **B** Heatmap of the top 100 DEGs

After differential analysis was performed by the “limma” R package with the threshold of FDR < 0.05 and |log_2_FC| > 1, the difference between 29 RP papillary tissues and 33 normal papillary tissues were presented in a volcano plot (Fig. 2A). A total of 116 DEGs were identified, including 73 up-regulated DEGs and 43 down-regulated DEGs. The 100 most up-regulated or down-regulated DEGs were visualized in a heatmap (Fig. 2B).

### Construction of a weighted co-expression network and identification of hub modules

**Fig. 3:**
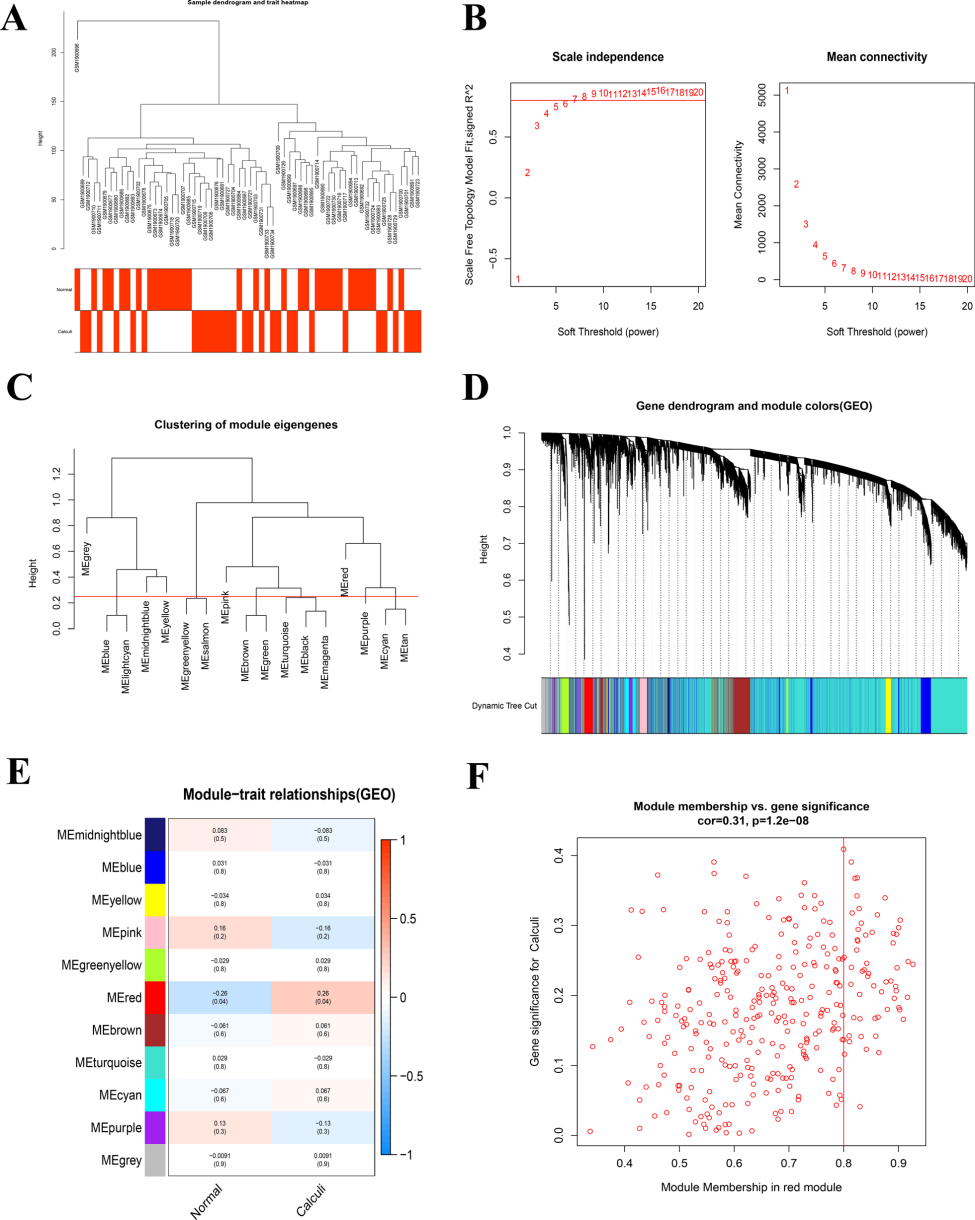
WGCNA for GSE73680. **A** Hierarchical clustering tree of 29 RP papillary tissues and 33 normal papillary tissues gene expression patterns. **B** Identification of power value. The red line represents R2 > 0.9 when the power value β is 7. **C** Module eigengene dendrogram presented the relationship of the modules generated by the clustering analysis. **D** Clustering dendrogram and merging of the gene co-expression modules. Each color represents one module. **E** Heatmap of the correlation between modules and clinical traits. The correlation coefficient and *P* value between the module and clinical traits are shown at the row-column intersection. **F** Scatter plot of module eigengenes in the red module, which is positively correlated with KSD

The “WGCNA” package was applied to explore the gene expression profiles in 29 RP papillary tissues and 33 normal papillary tissues. The samples were clustered hierarchically to remove outliers, but no samples were removed by the outliers check in this study (Fig. 3A). β = 7 was selected as soft-thresholding power value to make the scale-free R^2^ reach 0.9 and ensure a scale-free network (Fig. 3B). A total of 11 color modules were determined through average linkage clustering and dynamic tree cutting (Fig. 3C–D). The correlation between modules and clinical traits was presented in Fig. 3E, in which only the red module (r = 0.26, *P* = 0.04) represented a significantly positive correlation with stone disease. The number of genes contained in the red module is 324 and a significant correlation existed in the module membership and gene significance of the red module (Fig. 3F). Hence, the red module was selected as the key module for subsequent analysis.

### Functional and pathway enrichment analysis

**Fig. 4:**
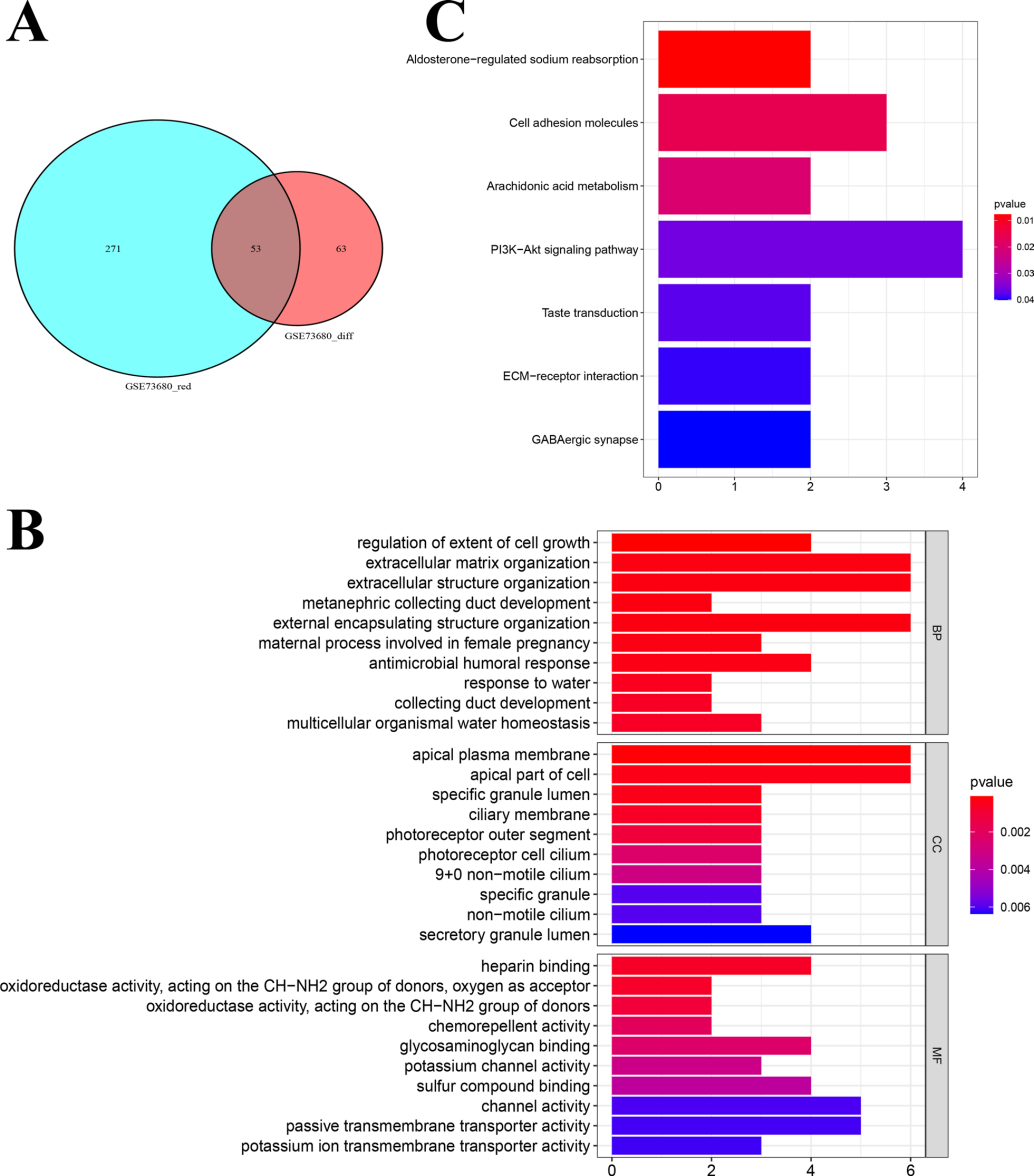
Functional and pathway enrichment analysis. **A** Venn plot showing the intersection between DEGs and the genes in the red module, and 53 genes were obtained. **B** GO enrichment analysis of the obtained 53 genes. **C** KEGG analysis of the obtained 53 genes

53 genes were obtained by taking the intersection of DEGs and genes in the red module (Fig. 4A). The selected genes were studied through GO and KEGG enrichment analysis to explore the biological functions associated with stone disease. The results of GO enrichment analysis were presented in Fig. 4B. Among the biological processes (BP) analysis, these genes were associated with regulation of extent of cell growth, extracellular matrix (ECM) organization, and extracellular structure organization. The term of apical plasma membrane was enriched in cellular component (CC) analysis, while heparin binding was enriched in molecular function (MF) analysis. KEGG enrichment analysis results indicated that aldosterone-regulated sodium reabsorption, cell adhesion molecules, arachidonic acid (AA) metabolism, PI3K-Akt signaling pathway, and ECM-receptor interaction were associated with these genes (Fig. 4C).

### Construction of PPI network and identification of hub genes

**Fig. 5:**
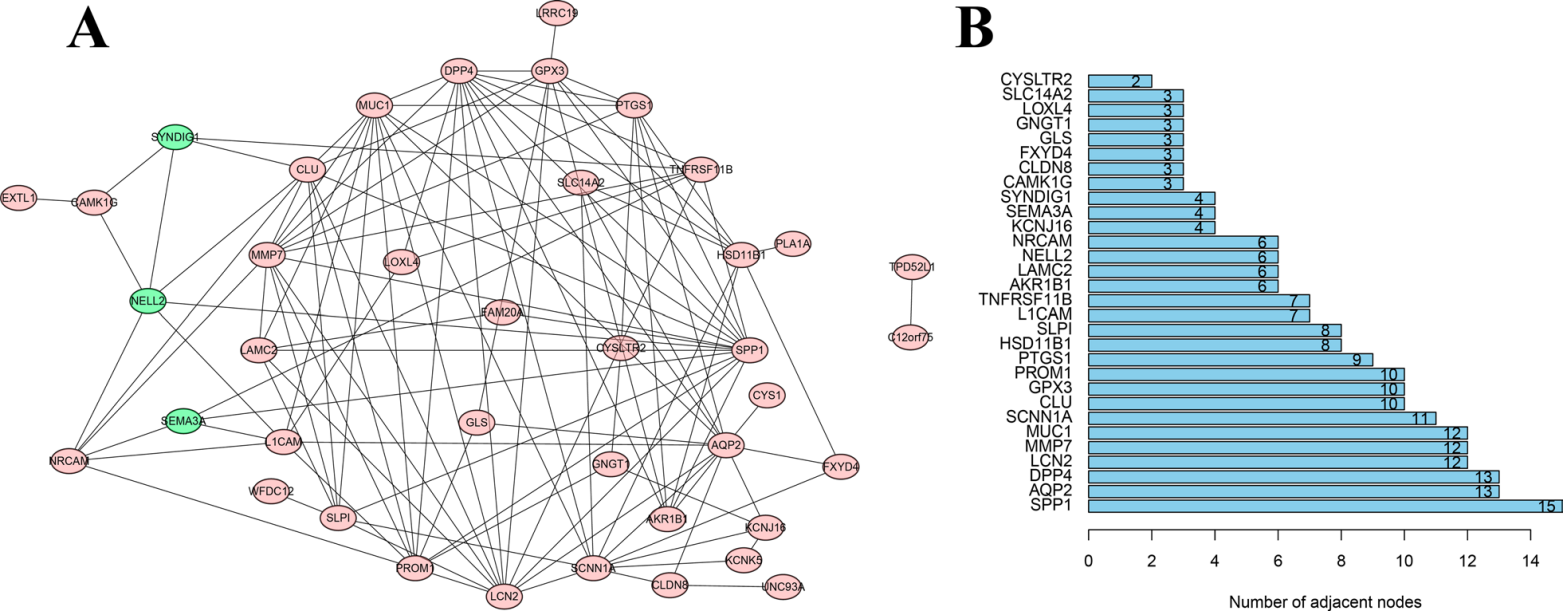
PPI network construction. **A** The PPI network was drawn using Cytoscape, and the network nodes represent proteins (red: up-regulated proteins and blue: down-regulated proteins). **B** Histogram of key genes

To further investigate the relationships among 53 genes at the protein level, the PPI network was constructed for candidate hub genes using Cytoscape according to STRING database. Up-regulated and down-regulated nodes were labeled with red and green, respectively (Fig. 5A). The top 30 genes with the largest number of adjacent nodes were screened as hub genes (28 up-regulated genes and 2 down-regulated genes), including SPP1, AQP2, DPP4, LCN2, MMP7, MUC1, SCNN1A, CLU, GPX3, PROM1, and so on (Fig. 5B). The detailed nodes and edges of the PPI network was listed in Additional file 2: Table S1.

### CIBERSORT analysis of immune infiltration

**Fig. 6:**
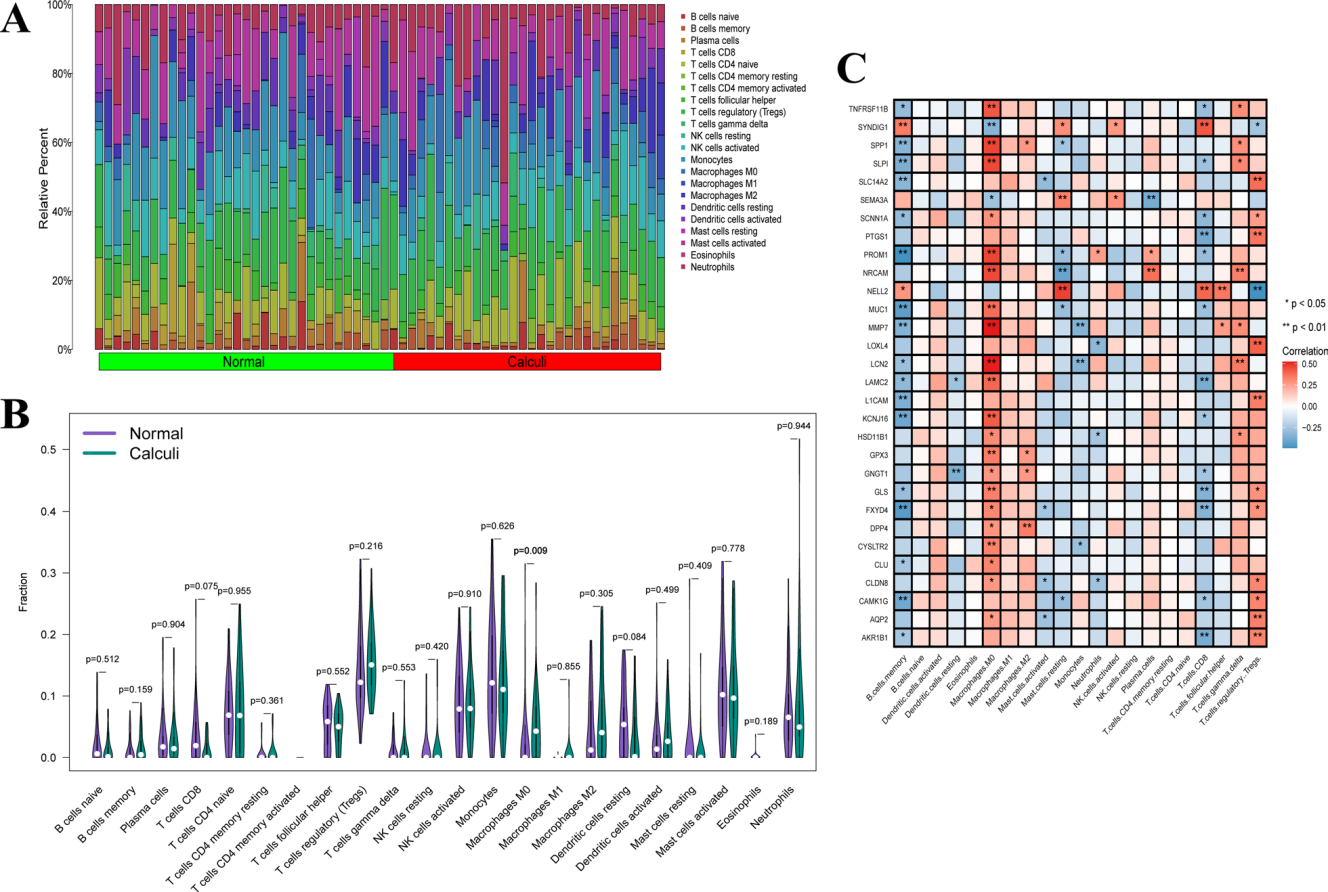
Immune infiltration levels based on CIBERSORT algorithm. **A** The composition ratio of 22 immune cell types. **B** Different proportions of immune cell subsets between RP papillary tissues and normal papillary tissues. **C** Display of correlations between hub genes and immune cells

CIBERSORT was performed to assess infiltrating levels of 22 immune cells in the RP papillary tissues and normal papillary tissues. The barplot displayed the relative composition ratio of 22 immune cells in all samples (Fig. 6A). Compared with control samples, RP papillary tissues harbored a higher proportion of macrophages M0, and the difference was statistically significant (*P* = 0.0093) (Fig. 6B). Correlation analysis showed that 21 up-regulated genes had a positive correlation with macrophages M0 and 2 down-regulated genes had a negative correlation with macrophages M0 (Fig. 6C).

### ssGSEA analysis of immune infiltration

**Fig. 7:**
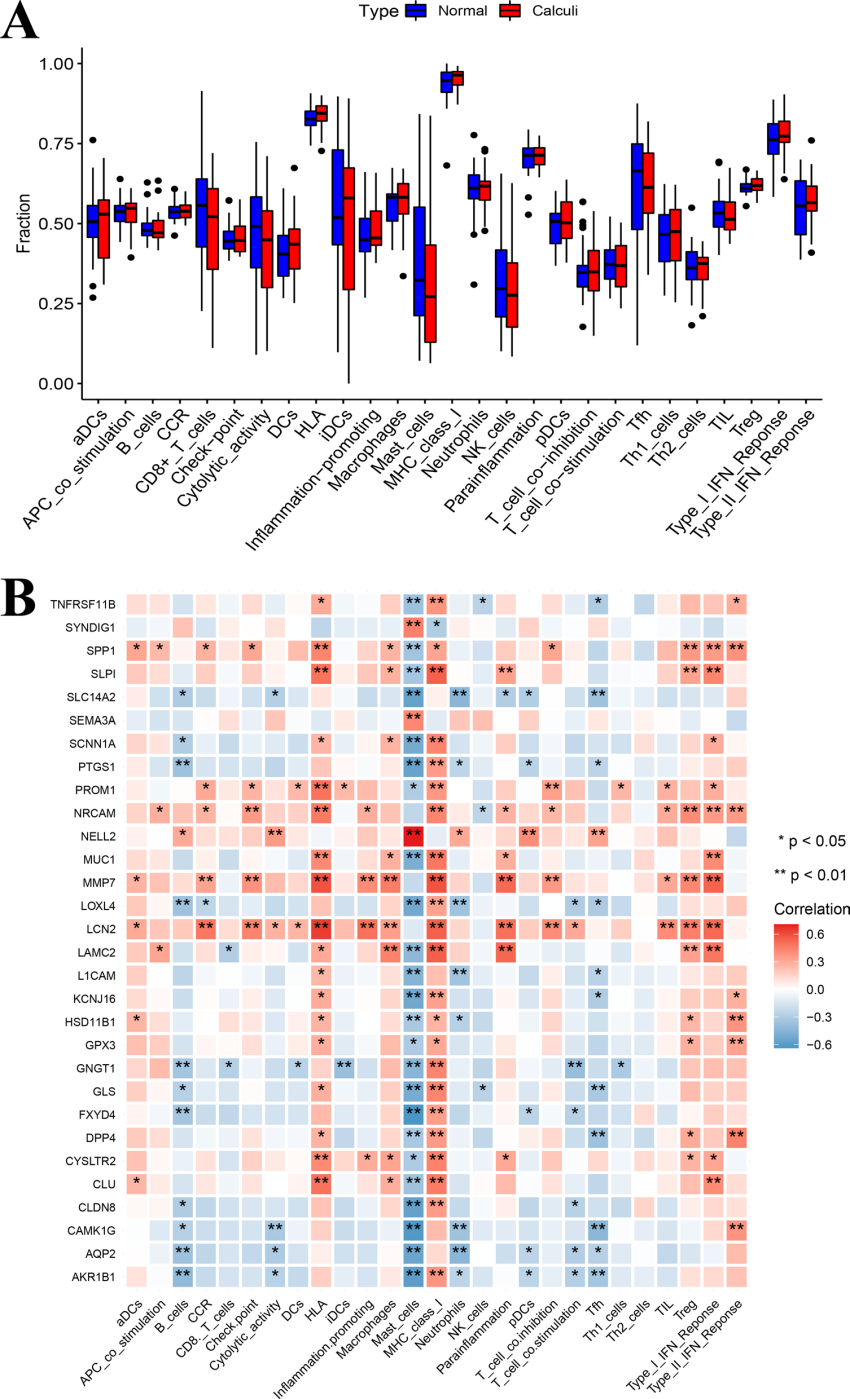
Immune infiltration levels based on ssGSEA. **A** Different proportions of 27 immune cells and immune-related functions between RP papillary tissues and normal papillary tissues. **B** Display of correlations between hub genes and immune cells and immune-related functions

The ssGSEA method was also applied to quantify the immune infiltration and the enrichment levels of 27 immune cells and immune-related functions in samples were obtained (Fig. 7A). However, there was no statistical significance between the two groups. Still, correlation analysis showed that 9 up-regulated genes had a positive correlation with macrophages (Fig. 7B). Combined with two methods, 9 up-regulated genes were strongly positively related to macrophages infiltration, including SPP1, LCN2, MMP7, MUC1, SCNN1A, CLU, SLP1, LAMC2, CYSLTR2.

### GSEA analysis based on SPP1 expression

**Fig. 8:**
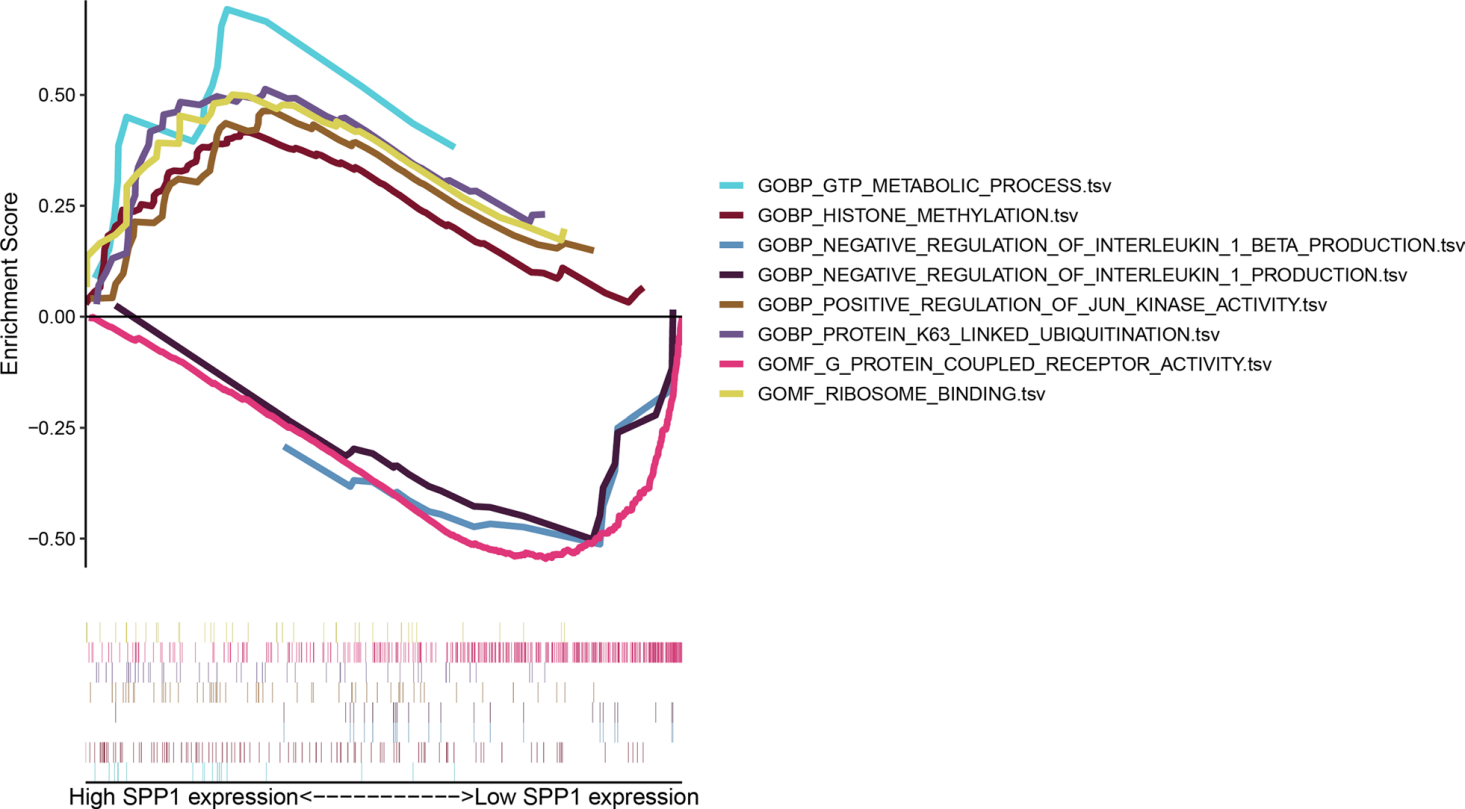
GSEA based on SPP1 expression levels

RP papillary tissues were divided into two subgroups based on the median expression of SPP1. GTP metabolic process, histone methylation, positive regulation of Jun kinase (JNK) activity, protein K63-linked ubiquitination, ribosome binding were significantly enriched in the high-SPP1 subgroup, while negative regulation of IL-1β production, negative regulation of IL-1 production, and G-protein coupled receptor activity were enriched in the low-SPP1 subgroup (Fig. 8).

## Discussion

The present study used multiple bioinformatic methods to identify KSD-related hub genes and explore the relationship between these genes and macrophage infiltration levels based on the GSE73680 dataset. A total of 116 DEGs were found by differential analysis. WGCNA was performed to construct a co-expression network and identify key modules associated with KSD, and the red module was selected as the key module. By taking the intersection of DEGs and genes in the red module, 53 genes were subjected to functional and pathway enrichment analyses and the PPI network construction. 30 hub genes were screened based on their numbers of adjacent nodes. By analyzing the immune characteristics of KSD patients via two algorithms, CIBERSORT analysis revealed a significantly increased proportion of M0 macrophages in RP tissues, while ssGSEA revealed no significant differences. Correlation analysis showed that SPP1, LCN2, MMP7, MUC1, SCNN1A, CLU, SLP1, LAMC2, and CYSLTR2 were positively correlated with macrophages infiltration. To our great interest, SPP1 had the highest number of edges and was selected as the top hub gene for further GSEA analysis.

GO enrichment analysis indicated that genes were enriched in ECM organization and extracellular structure organization. KEGG enrichment analysis suggested that genes were bound up with cell adhesion molecules and ECM-receptor interaction. Collagen is an important component of the ECM, and RPs consist of CaP crystals mixed with membrane-bound vesicles, collagen fibers, and other components of the ECM [23]. In RP theory, interstitial apatite deposits must grow outwards and reach the renal papillary surface to contact the pelvic urine for further CaOx deposition [24]. Such a breach must require the involvement of ECM and extracellular structure reconstruction and remodeling. Based on microscopic and analytical studies, Khan et al. first proposed that matrix vesicles promote CaP crystals formation and these calcified deposits progress through the mineralization of collagen and other components of the ECM, leading to the expansion of RPs [23]. In addition, the pathway of aldosterone-regulated sodium reabsorption and AA metabolism were also significantly enriched. A recent study has revealed the influences of aldosterone and intra-vascular volume on calcium homeostasis and urinary calcium levels, suggesting the potential role of this pathway in KSD [25]. Baggio et al. found higher contents of AA and prostaglandin E2 (PGE2) in plasma, higher urinary calcium excretion, as well as intestinal calcium absorption in idiopathic calcium stone formers compared to healthy controls [26]. Increased AA may activate the intestinal and renal transport of oxalate to induce hyperoxaluria [27]. PGE2, a bioactive lipid generated from AA, could increase urinary calcium excretion by affecting renal tubular function and increasing intestinal calcium absorption [27]. Further studies are needed to clarify the potential implication of these pathways in KSD.

Water et al. first reported the infiltration of macrophages around the interstitial crystals in human kidney papillae [28]. Since then, a growing number of studies have confirmed the involvement of macrophages in stone formation and development, most of which have focused on the role of macrophages polarization. Generally, M0 macrophages can polarize into two main phenotypes, pro-inflammatory M1 phenotype and anti-inflammatory M2 phenotype, depending on local microenvironment [29]. In human kidney tissues, stone formers showed an increased gene expression associated with M1 phenotype and a decreased gene expression associated with M2 phenotype compared with controls [30]. Monocytes can be differentiated into M1 macrophages under CaOx crystals stimulation and M1 macrophages promote crystal deposition and accelerate stone development via enhancing tissue damage and renal inflammation [30–32]. By contrast, M2 macrophages are involved in the suppression of stone formation due to potent crystal phagocytic ability [30, 33, 34]. Thus, there has been an increasing interest in altering the macrophages phenotypes as therapeutic targets.

Given the importance of macrophages in KSD, immune infiltration was assessed in the dataset by applying two independent analytical methods. Although there were no statistically significant differences in M1 and M2 macrophages infiltration levels between the two groups, CIBERSORT analysis revealed that the proportion of M0 macrophages in RP papillary tissues was higher than that in normal papillary tissues. Non-polarized macrophages were designated as M0 phenotype. In vitro studies have demonstrated that M0 macrophages were able to internalize and eliminate COM crystals via phagocytosis [35, 36]. M0 macrophages could also affect the function of other cells. Zou et al. found that renal tubular epithelial cells (RTECs) showed increased COM crystal adhesion and induced more expression of inflammatory cytokines of SPP1, MCP-1, and TNF-a when they were co-cultured with M0 macrophages [37]. Exosomes derived from COM-treated M0 macrophages can stimulate IL-8 secretion from RTECs and monocytes, activate monocytes and neutrophils migration, and enhance macrophages phagocytic activity [38, 39]. Moreover, these exosomes have higher binding capacity to COM crystals due to exosomal membrane fragility, which helps crystal invasion through ECM in the renal interstitium [38]. Our results further confirmed that macrophages serve an important role during the stone formation process.

PPI network was constructed and 30 genes were selected as hub genes according to their numbers of adjacent nodes. Furthermore, 9 hub genes were strongly positively related to macrophages infiltration based on two methods, including SPP1, LCN2, MMP7, MUC1, SCNN1A, CLU, SLP1, LAMC2, CYSLTR2. SPP1 had the highest number of edges and was chosen as the top hub gene. In the human kidneys, SPP1 is expressed in the thick ascending limbs of the loop of Henle, collecting ducts, and urine [40]. Some studies have reported decreased urinary excretion of SPP1 in stone formers than controls [41, 42], while others showed increased excretion or no difference [43, 44]. Still, SPP1 is identified as one of the most important organic matrix components in calcium stones and numerous studies showed that SPP1 expression was increased in animal models [45]. In vitro studies found that SSP1 was able to inhibit CaOx crystal nucleation, growth, and aggregation [46–48]. However, the role of SPP1 in crystal adhesion is controversial. Wesson et al. observed that SPP1 might favor CaOx dehydrate (COD) formation rather than CaOx monohydrate (COM), and COD is less adherent to RTECs, which could reduce crystal attachment [49]. By contrast, Yamate et al. considered SPP1 as a promoter of stone formation because of increased crystal adhesion and deposition in its presence [50–52]. The reason for two completely different actions may lie in the fact that SPP1 has two forms: free and immobilized SPP1 play inhibitory and supportive roles in stone formation, respectively [53]. Notably, it has been reported that some polymorphisms in the OPN gene may predispose to stone disease [54, 55]. In addition, SPP1 is a significant chemical attractant for macrophages, dendritic cells, and T cells. RTECs stimulated by crystal deposition could secrete SPP1 to induce macrophage migration and phagocytosis [56].

GSEA analysis showed that positive regulation of JNK activity was significantly enriched in the high-SPP1 subgroup. Several studies have demonstrated that the JNK pathway was activated in RTECs after high oxalate or calcium exposure and activation of the JNK pathway could induce SPP1 expression and crystal deposition [53, 57, 58]. This is also supported by our results. In addition, negative regulation of IL-1β production was significantly enriched in the low-SPP1 subgroup. Mulay et al. have found that CaOx crystals could activate intrarenal dendritic cells to secrete IL-1β via the NLRP3 inflammasome pathway and lead to renal damage [59]. Hence, IL-1β production might aggravate the progression of KSD. The low-SPP1 subgroup may have a lower infiltration of immune cells, which leads to a decreased production of IL-1β and attenuation of kidney injury.

In this study, the key modules and hub genes related to KSD were screened, their biological functions and pathways were identified, and the associations between hub genes and macrophages were also studied, which shed light on the potential pathogenic mechanism of KSD and present an avenue for therapeutic exploration. Specifically, the findings suggest that SPP1 plays a pivotal role in KSD and the interaction between SPP1 and macrophages may be crucial for stone formation. Therefore, SPP1 could serve as a biomarker for the early diagnosis and a target for the treatment of KSD, and inhibition of SPP1 might modulate the phenotype of macrophages to protect against stone formation.

This study has some limitations. First, there are few datasets about KSD in GEO database. Thus, an external validation set to verify the accuracy is missing. Second, in vivo and in vitro studies need to be conducted to investigate potential mechanisms of real hub genes and macrophages for future clinical translation.

## Conclusion

Our research uses WGCNA, combined with immune infiltration analysis and correlation analysis to identify the hub genes in KSD, which provides further insights into potential therapeutic targets for KSD. As the top hub gene, SPP1 is widely connected with other hub genes, and a great number of studies have confirmed the role of SPP1 in stone formation and development. However, additional studies are needed to elucidate the role the interactions between SPP1 and macrophages play in KSD.

## Supplementary Information

**Additional file 1.** Code used to perform the multiple bioinformatic analysis.

**Additional file 2.** Detailed nodes and edges of the PPI network.

## Data Availability

All raw and processed data are freely available from GEO database (https://www.ncbi.nlm.nih.gov/geo/).

## Abbreviations

KSD: Kidney stone disease
DEGs: Differentially expressed genes
WGCNA: Weighted gene co-expression network analysis
GO: Gene Ontology
KEGG: Kyoto Encyclopedia of Genes and Genomes
PPI: Protein–protein interaction
CaOx: Calcium oxalate
CaP: Calcium phosphate
RP: Randall’s plaque
GSEA: Gene set enrichment analysis
ssGSEA: Single sample gene set enrichment analysis
BP: Biological processes
CC: Cellular component
MF: Molecular function
COM: Calcium oxalate monohydrate
COD: Calcium oxalate dehydrate
RTECs: Renal tubular epithelial cells
FDR: False discovery rate
FC: Fold change
TOM: Topological overlap measure
ECM: Extracellular matrix
JNK: Jun kinase
AA: Arachidonic acid
