# Supplementary material for: Identification of the pivotal role of SPP1 in kidney stone disease based on multiple bioinformatics analysis
Source: BMC Med Genomics. 2022 Jan 11;15:7. doi: 10.1186/s12920-022-01157-4 (PMC8751247; doi:10.1186/s12920-022-01157-4)
Supplement: Supplementary file 2 — Additional file 2. Detailed nodes and edges of the PPI network. [file 12920_2022_1157_MOESM2_ESM.docx]

Table S1. Detailed nodes and edges of the PPI network.

| Edges | | Combined scores |
| --- | --- | --- |
| Node1 | Node2 |  |
| AKR1B1 | AQP2 | 0.398 |
| AKR1B1 | SPP1 | 0.2 |
| AKR1B1 | HSD11B1 | 0.288 |
| AKR1B1 | PTGS1 | 0.304 |
| AKR1B1 | GPX3 | 0.313 |
| AKR1B1 | DPP4 | 0.404 |
| AQP2 | DPP4 | 0.156 |
| AQP2 | GLS | 0.158 |
| AQP2 | MUC1 | 0.16 |
| AQP2 | L1CAM | 0.171 |
| AQP2 | PTGS1 | 0.198 |
| AQP2 | KCNJ16 | 0.232 |
| AQP2 | CLDN8 | 0.266 |
| AQP2 | FXYD4 | 0.278 |
| AQP2 | LCN2 | 0.321 |
| AQP2 | CYS1 | 0.49 |
| AQP2 | SCNN1A | 0.637 |
| AQP2 | SLC14A2 | 0.796 |
| C12orf75 | TPD52L1 | 0.241 |
| CAMK1G | NELL2 | 0.166 |
| CAMK1G | SYNDIG1 | 0.175 |
| CAMK1G | EXTL1 | 0.194 |
| CLDN8 | UNC93A | 0.222 |
| CLDN8 | SCNN1A | 0.177 |
| CLU | MMP7 | 0.195 |
| CLU | NELL2 | 0.158 |
| CLU | SYNDIG1 | 0.16 |
| CLU | SLPI | 0.187 |
| CLU | PROM1 | 0.215 |
| CLU | NRCAM | 0.22 |
| CLU | MUC1 | 0.239 |
| CLU | GPX3 | 0.325 |
| CLU | SPP1 | 0.583 |
| CLU | LCN2 | 0.648 |
| CYSLTR2 | GNGT1 | 0.6 |
| CYSLTR2 | PTGS1 | 0.218 |
| DPP4 | MMP7 | 0.16 |
| DPP4 | LOXL4 | 0.184 |
| DPP4 | TNFRSF11B | 0.16 |
| DPP4 | SCNN1A | 0.175 |
| DPP4 | PTGS1 | 0.161 |
| DPP4 | GPX3 | 0.179 |
| DPP4 | MUC1 | 0.22 |
| DPP4 | LCN2 | 0.235 |
| DPP4 | HSD11B1 | 0.316 |
| DPP4 | SPP1 | 0.323 |
| DPP4 | PROM1 | 0.478 |
| FAM20A | LAMC2 | 0.249 |
| FAM20A | SPP1 | 0.369 |
| FXYD4 | SCNN1A | 0.297 |
| FXYD4 | HSD11B1 | 0.215 |
| GLS | GPX3 | 0.21 |
| GLS | PROM1 | 0.219 |
| GNGT1 | PROM1 | 0.166 |
| GNGT1 | KCNJ16 | 0.617 |
| GPX3 | MMP7 | 0.191 |
| GPX3 | PTGS1 | 0.266 |
| GPX3 | HSD11B1 | 0.229 |
| GPX3 | LCN2 | 0.202 |
| GPX3 | LRRC19 | 0.18 |
| GPX3 | SPP1 | 0.228 |
| HSD11B1 | SLC14A2 | 0.452 |
| HSD11B1 | PLA1A | 0.165 |
| HSD11B1 | SCNN1A | 0.41 |
| HSD11B1 | PTGS1 | 0.18 |
| KCNJ16 | KCNK5 | 0.445 |
| KCNJ16 | SCNN1A | 0.359 |
| KCNK5 | SCNN1A | 0.219 |
| L1CAM | LOXL4 | 0.175 |
| L1CAM | SEMA3A | 0.541 |
| L1CAM | NELL2 | 0.244 |
| L1CAM | MUC1 | 0.293 |
| L1CAM | NRCAM | 0.304 |
| L1CAM | PROM1 | 0.394 |
| LAMC2 | MMP7 | 0.926 |
| LAMC2 | SPP1 | 0.159 |
| LAMC2 | MUC1 | 0.16 |
| LAMC2 | SLPI | 0.165 |
| LAMC2 | LCN2 | 0.177 |
| LCN2 | MMP7 | 0.439 |
| LCN2 | TNFRSF11B | 0.244 |
| LCN2 | SLPI | 0.756 |
| LCN2 | SCNN1A | 0.527 |
| LCN2 | PROM1 | 0.178 |
| LCN2 | MUC1 | 0.392 |
| LCN2 | SPP1 | 0.614 |
| LOXL4 | TNFRSF11B | 0.158 |
| MMP7 | PTGS1 | 0.159 |
| MMP7 | NRCAM | 0.171 |
| MMP7 | TNFRSF11B | 0.262 |
| MMP7 | SLPI | 0.269 |
| MMP7 | PROM1 | 0.353 |
| MMP7 | MUC1 | 0.526 |
| MMP7 | SPP1 | 0.968 |
| MUC1 | SLPI | 0.354 |
| MUC1 | SCNN1A | 0.232 |
| MUC1 | PTGS1 | 0.17 |
| MUC1 | SPP1 | 0.451 |
| MUC1 | PROM1 | 0.456 |
| NELL2 | SYNDIG1 | 0.161 |
| NELL2 | NRCAM | 0.411 |
| NELL2 | SPP1 | 0.155 |
| NRCAM | SEMA3A | 0.72 |
| NRCAM | PROM1 | 0.168 |
| PROM1 | SPP1 | 0.457 |
| PTGS1 | SPP1 | 0.2 |
| SCNN1A | SLC14A2 | 0.502 |
| SCNN1A | SLPI | 0.162 |
| SEMA3A | SPP1 | 0.241 |
| SEMA3A | TNFRSF11B | 0.249 |
| SLPI | WFDC12 | 0.185 |
| SLPI | SPP1 | 0.257 |
| SPP1 | TNFRSF11B | 0.776 |
| SYNDIG1 | TNFRSF11B | 0.151 |
